# The therapeutic role of Cannabidiol in mental health: a systematic review

**DOI:** 10.1186/s42238-019-0012-y

**Published:** 2020-01-02

**Authors:** Rabia Khan, Sadiq Naveed, Nadeem Mian, Ania Fida, Muhammad Abdur Raafey, Kapil Kiran Aedma

**Affiliations:** 1 Dow University of Health Science, Karachi, Pakistan; 2 Psychiatry and Behavioral Sciences, Kansas University Medical Center, 3901 Rainbow Blvd, Kansas City, KS KS 66160 USA; 3 PICACS Clinic, Bothell, WA USA; 4 King Edward Medical University, Lahore, Pakistan; 5 Child and Adolescent Psychiatrist, KVC Hospitals, Kansas, USA

**Keywords:** Cannabidiol (CBD), Nabiximols, Schizophrenia, Cannabis, Withdrawal, Dependence, Autism spectrum disorder (ASD), Attention deficit hyperactivity disorder (ADHD), Post-traumatic stress disorder (PTSD), Tourette syndrome, Bipolar disorder

## Abstract

**Background:**

The therapeutic application of cannabidiol (CBD) is gaining interest due to expanding evidence for its use.

**Objective:**

To summarize the clinical outcomes, study designs and limitations for the use of CBD and nabiximols (whole plant extract from *Cannabis sativa* L. that has been purified into 1:1 ratio of CBD and delta-9-tetrahydrocannabinol) in the treatment of psychiatric disorders.

**Materials and method:**

A systematic review was conducted including case reports, case series, open-label trials, non-randomized and randomized controlled trials (RCTs). The search resulted in 23 relevant studies on CBD and nabiximols in the treatment of a wide range of psychiatric disorders. The quality of evidence was judged by using the Oxford Centre for Evidence-Based Medicine 2011 Levels of Evidence that ranges from Level 1 to Level 5 based on the quality and study design. These levels of evidence help in grading the recommendations, including Grade A (strong), Grade B (moderate), Grade C (weak), and Grade D (weakest).

**Results:**

CBD and CBD-containing compounds such as nabiximols were helpful in alleviating psychotic symptoms and cognitive impairment in patients with a variety of conditions, and several studies provided evidence of effectiveness in the treatment of cannabis withdrawal and moderate to severe cannabis use disorder with Grade B recommendation. There is Grade B recommendation supporting the use of CBD for the treatment of schizophrenia, social anxiety disorder and autism spectrum disorder (ASD), and attention deficit hyperactivity disorder (ADHD). Grade C recommendation exists for insomnia, anxiety, bipolar disorder, posttraumatic stress disorder, and Tourette syndrome. These recommendations should be considered in the context of limited number of available studies.

**Conclusion:**

CBD and CBD-containing compounds such as nabiximols were helpful in alleviating symptoms of cannabis-related disorders, schizophrenia, social anxiety disorder, and comorbidities of ASD, and ADHD with moderate recommendation. However, there is weaker evidence for insomnia, anxiety, bipolar disorder, posttraumatic stress disorder, and Tourette syndrome. The evidence for the use of CBD and CBD-containing compounds for psychiatric disorders needs to be explored in future studies, especially large-scale and well-designed RCTs.

## Introduction

*Cannabis sativa*, a species of cannabis plant, is well known to humankind, with its earliest use in ancient Chinese culture dating as far back as 2700 B.C. (Zuardi, 2006). The use of medical cannabis in China was reported in the world’s oldest pharmacopoeia (Martin et al., 1999). However, interest in the role of cannabis flourished in the late twentieth century after the recognition of an endogenous cannabinoid system in the brain (Zuardi, 2006; Martin et al., 1999). More recently, research has centered on the description and cloning of specific receptors and the therapeutic effects of medical cannabis, and different cannabinoids in the cannabis plant have gained interest (Martin et al., 1999). Recent studies have focused on the therapeutic role of medical cannabis in different disorders. As a result, there is a growing need to summarize and review the evidence for its therapeutic and adverse effects as an aid to public health policy development, and to provide direction and impetus to pharmaceutical research in this field.

The cannabis plant has more than 140 cannabinoid compounds, with Δ9-tetrahydrocannabinol (Δ9-THC) and cannabidiol (CBD) attracting significant interest (Citti et al., 2018). Δ9-THC is the primary psychoactive ingredient, and CBD is a non-intoxicating ingredient (Zuardi, 2006; Citti et al., 2018). Evidence from preclinical studies suggested that CBD had potential therapeutic benefits ranging from antiinflammatory to neuroprotective, antipsychotic, analgesic, anticonvulsant, antiemetic, antioxidant, antiarthritic, and antineoplastic properties; for a review, see (Pertwee, 2006). CBD has several receptors and molecular targets. This compound antagonizes the action of CB_1_ and CB_2_ receptor agonist (Blessing et al., 2015; Peres et al., 2018). The CB_1_ and CB_2_ receptors are coupled negatively through G-proteins to adenylate cyclase and positively to mitogen-activated protein kinase (Pertwee, 2006). In addition to CB_1_ and CB_2_ receptor activity, CBD is an agonist of vanilloid receptor TRPV_1_. It also acts as an agonist of serotonin receptor 5-hydroxytryptamine (5-HT_1A_), an antagonist of G-protein-coupled receptor GPR55, and an inverse agonist of GPR3, GPR6, and GPR12 (Peres et al., 2018). Data from single-photon emission computed tomography showed CBD to exert anxiolytic effects by acting on paralimbic and limbic pathways (Crippa et al., 2011). The agonist effect of CBD on 5-HT_1A_ also supports its anxiolytic and antidepressant properties (Russo et al., 2005). CBD inhibits enzymatic hydrolysis and anandamide uptake through its agonist action on CB_1_, CB_2_, and TRPV_1_ receptors (Peres et al., 2018). In addition, CBD indirectly enhances endogenous anandamide signaling by inhibiting the intercellular degradation of anandamide (Leweke et al., 2012). This endogenous neurotransmitter exerts antipsychotic effects in patients with schizophrenia (Leweke et al., 2012).

The pharmacokinetic profile of CBD has been extensively explored in the existing literature. A recently published systematic review of the pharmacokinetics of CBD found that the area under curve (AUC0 − t) and maximum serum concentration (Cmax) occurs between 1 and 4 h (Millar et al., 2018). The AUC_0 − t_ and Cmax reach maximum values faster after smoking or inhalation compared to oral or oromucosal routes. Bioavailability was 31% after smoking, but no other studies reported the absolute bioavailability of CBD after other routes in humans. The half-life of CBD ranges between 1.4 and 10.9 h after oromucosal spray and 2–5 days after chronic oral administration (Millar et al., 2018). Fed states and lipid formulations increase Cmax (Millar et al., 2018). The bioavailability of oral CBD ranges between 11 and 13%, compared to 11 to 45% (mean 31%) via inhalation (Scuderi et al., 2009). CBD is well-tolerated, yet despite a relatively lower risk of drug–drug interactions, it should be used cautiously in combination with drugs metabolized by the CYP3A4 and CYP2C19 pathways, and the substrates of UDP-glucuronosyltransferases UGT1A9 and UGT2B7 (Millar et al., 2018). The clinical relevance of these interactions needs to be explored in future studies (Brown & Winterstein, 2019).

Dronabinol and nabilone are synthetic in origin, whereas nabiximols is plant-based (Papaseit et al., 2018). The percentage of THC and its ratio to CBD (THC/CBD ratio) defines the potency and psychoactive effects of a given formulation (Papaseit et al., 2018). Those with higher CBD/Δ9-THC ratios have euphoric, anxiolytic, and relaxing effects, whereas lower CBD/Δ9-THC ratios have sedative properties (Papaseit et al., 2018). Nabiximols, a CBD-containing compound, contains Δ9-THC and CBD at a 1:1 ratio (Papaseit et al., 2018). The Food and Drug Administration has approved Epidiolex® (an oral formulation of CBD) for two forms of childhood seizures (Lennox–Gastaut syndrome and Dravet syndrome) in children 2 years of age and older (Papaseit et al., 2018).

Previous efforts to synthesize the evidence for medical cannabis use in patients with psychiatric disorders have been published (Hoch et al., 2019; Lowe et al., 2019). For example, Hoch et al. conducted an excellent systematic review that summarized four systematic reviews and 14 randomized controlled trials (RCTs), but did not consider non-clinical trial evidence (case reports and case series) (Hoch et al., 2019). A review by Mandolini et al. recently summarized the clinical findings from 14 studies of psychiatric disorders, but these authors did not provide information about nabiximols (Mandolini et al., 2018). In contrast to the review articles noted above, the present article aims to provide a more comprehensive review of the use of CBD and CBD-containing compounds such as nabiximols to treat psychiatric disorders. The present review included studies focused on schizophrenia, cannabis-related disorders, attention deficit hyperactivity disorder (ADHD), comorbidities in autism spectrum disorder (ASD), social anxiety disorder (SAD), other anxiety disorders, insomnia, bipolar disorder, post-traumatic stress disorder (PTSD), psychosis in Parkinson’s disease, and Tourette syndrome. This article broadly reviews the efficacy, safety, and psychiatric benefits of CBD and CBD-containing compounds (nabiximols). We distinguish clearly here between the clinical findings for CBD and nabiximols, as the latter also contains THC.

## Methods

### Eligibility criteria

The main inclusion criterion was studies of the psychiatric use of CBD and CBD-containing compounds such as nabiximols. Only case reports, case series, retrospective chart reviews, open-label trials, and RCTs were considered. All books, conference papers, theses, editorials, review articles, metaanalyses, in-vitro studies, laboratory studies, animal studies, studies of participants without psychiatric disorders, and abstract-only articles were excluded. No restrictions on language, country, publication year, or patients’ age, gender, or ethnicity were applied.

### Search strategy

Eight electronic databases were searched on October 28th, 2018: PubMed, Scopus, Web of Science, POPLINE, New York Academy of Medicine Grey Literature Report, PsycINFO, Psycarticles, and CINAHL. The following search strategy was used in all cases: (CBD OR Cannabi* OR nabiximols) AND (psychiat* OR Depress* OR Anxiety OR Psycho* OR schizo* OR Bipolar OR Substance OR ADHD OR Attention OR Autism) AND (treatment). The manual search of references of included studies was performed by four independent reviewers.

### Study selection

The search results from the eight databases were imported to Endnote v. 7 (Thompson Reuters, CA, USA) to remove any duplicates. Four independent reviewers (RK, NM, AF, MAF) screened the titles and abstracts (when available), followed by full-text screening of each included article with the predetermined eligibility criteria. All articles included after full-text screening were then searched manually. Discrepancies were resolved by consensus through discussion among reviewers, or with guidance from a third reviewer (SN).

### Data extraction and grading

The data were extracted independently by the authors, and were cross-checked by discussion among the four reviewers (RK, NM, AF, MAF), with guidance from the senior author (SN) in case of discrepancy. The data were categorized as pertaining to target diagnosis, study design, sample size, duration of the trial, age range, dose ranges, measurement scales, clinical outcomes, study limitations, and common side effects.

The Oxford Centre for Evidence-Based Medicine 2011 Levels of Evidence was used to grade the quality of evidence (OCEBM, 2019). Level 1 evidence is for systematic review of RCTs or individual RCT of narrow confidence interval, Level 2 for cohort studies or systematic review of cohort studies, Level 3 for case-control studies or systematic review of case-control studies, and Level 4 for case-series for studies focused on therapy, prevention, etiology and harm (OCEBM, 2019). These levels of evidence are used to generate Grades of Recommendation. Grade A is for consistent level 1 studies, Grade B for consistent level 2 or 3 studies or extrapolations from level 1 studies, and Grade C for level 4 studies or extrapolations from level 2 or 3 studies. Grade D is ranked for level 5 evidence or inconsistent or inclusive studies of any level (OCEBM, 2019).

## Results & discussion

**Fig. 1:**
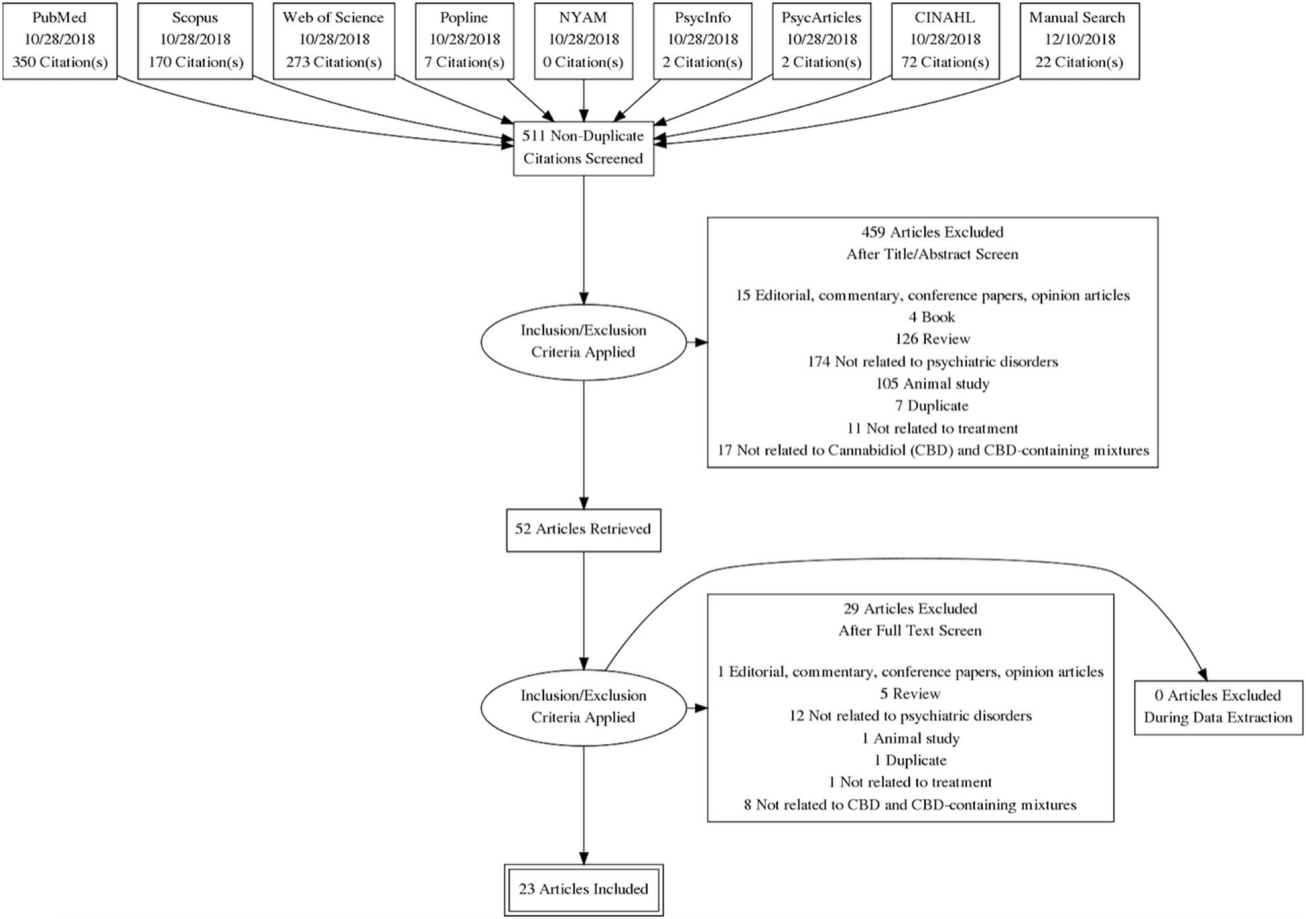
PRISMA Flow Diagram

The search of eight electronic databases and our manual screening method generated 511 results. After the removal of duplicates, titles and abstracts were screened, resulting in the exclusion of 459 articles. Full-text screening of 52 articles was performed, and 23 articles meeting the inclusion criteria were analyzed. Figure 1 summarizes the screening process.

Of the 23 articles, there were eight RCTs, one clinical trial, four open-label trials, one retrospective chart review, seven case reports, and two case series, comprising a total patient population of 526. The studies focused on CBD and nabiximols use in the treatment of schizophrenia, cannabis-related disorders, ADHD, ASD and comorbidities, anxiety, insomnia, SAD, bipolar disorder, PTSD, psychosis in Parkinson’s disease, and Tourette syndrome. No studies of substance use disorders other than cannabis use were identified. In this review article, the authors have used DSM-5 terminologies for most of the disorders except for DSM-IV-Text Revised terminology of substance dependence. A comparable DSM-5 terminology of moderate-severe substance use disorder was used in this case.

### Qualitative synthesis of eligible studies

#### Schizophrenia and psychosis in Parkinson’s disease

There were three RCTs (164 patients), one clinical trial (27 patients), one case series (three patients), one case report for schizophrenia, and one open-label trial (six patients) for psychosis in Parkinson’s disease (Table 1) (Leweke et al., 2012; Hallak et al., 2010; Boggs et al., 2018; McGuire et al., 2018; Zuardi et al., 2006; Zuardi et al., 1995; Zuardi et al., 2009). Of the seven studies, level 2 evidence was found in three RCTs, level 3 evidence in two clinical trial, and level 4 evidence in one case report and one case series (OCEBM, 2019). Since most of the studies were from level 2 and level 3 evidence, there is Grade B recommendation for schizophrenia. The dose of CBD in these studies ranged from 200 to 1500 mg daily. The highest dose was titrated to 1500 mg daily as reported by Zuardi and colleagues (Zuardi et al., 1995). Irrespective of the study design, three studies reported that CBD alleviated psychotic symptoms and cognitive impairment in patients with chronic cannabis use and Parkinson’s disease (Leweke et al., 2012; Zuardi et al., 1995; Zuardi et al., 2009), while only two RCTs and one clinical trial provided evidence for the effectiveness of CBD among patients with schizophrenia, albeit with mixed results (Leweke et al., 2012; McGuire et al., 2018; Zuardi et al., 2009).

**Table 1:** Studies of CBD use in the treatment of schizophrenia and psychosis in Parkinson’s disease and levels of evidence (1 to 5)*

Author	Diagnosis	Pharmacological agent	Study design	Strength of evidence*	Group (n)	Duration	Age range (years)	Dose range (mg)	Scales to measure the clinical outcome	Clinical outcome	Common side effects	Reference number
Hallak al., 2010	Schizophrenia	CBD	RCT	Level 2	CBD 300 mg = 9 CBD 600 mg = 8 Placebo = 10	1 month	> 18	CBD = 300 or 600 mg	SCWT	- The SCWT and skin conductance were recorded at baseline and 1 month after the initial test. Patients received CBD or placebo before the test. - In the first session, there was significant SCWT effect on electrodermal response factor only (F1,16 = 5.98; *p* < 0.05) related to time taken to complete board I. - The mean time required for the responsive group was 77.8 (SEM = 11.7) and for the non-responsive it was was 119.7 (SEM = 12.3). - In the second assessment, a significant effect for number of errors on board II (F2,16 = 6.027; *p* = 0.014). The group that received CBD 600 mg had a higher score compared to the other two. - SCWT score improved in the placebo and 300 mg group, but the improvement was smaller in the 600 mg CBD group. The improvement in participants given CBD 600 mg was smaller due to sedation.	No side effects were reported.	18
Leweke et al., 2012	Schizophrenia	CBD	RCT	Level 2	CBD = 20 Amisulpride = 19	4 weeks	18–50	- Participants were started on 200 mg/day of CBD or amisulpride - The dose was increased by 200 mg/day in the 1st week. - The total dose was 200 mg four times daily (800 mg/day)	BPRS, PANSS, EPS, serum prolactin, body weight	- Patients in both groups reported a comparable improvement in PANSS and BPRS (1.0, 95% confidence interval 12.6 to 14.6, *P* = 0.884. - CBD inhibited FAAH activity and increased intrinsic anandamide signaling, resulting in antipsychotic properties. There was a a statistically significant association between higher anandamide levels and decrease in psychotic symptoms in patients treated with cannabidiol (*P* = .0012)	Treatment with CBD was associated with lower risk of EPS, less weight gain, and a lower increase in prolactin level - a predictor of galactorrhea and sexual dysfunction.	9
Boggs et al., 2018	Schizophrenia	CBD	RCT	Level 2	CBD = 18 Placebo = 18	6 weeks	18–65	CBD = 600 mg/day	MCCB, PANSS	- For MCCB Composite score, there was no effect of drug or time, but a significant drug × time effect was observed (F (1, 32) = 5.94; *p* = 0.02). - There was only improvement in placebo-treated subjects time (F (1, 32) = 4.84; *p* = 0.03). - Lack of improvement in psychotic symptoms on PANSS (F (3, 101) = 1.66; p = 0. 18).	Mild sedation was reported in 20% of participants compared to 5% in placebo.	22
McGuire et al., 2018	Schizophrenia	CBD	RCT	Level 2	CBD = 43 Placebo = 45	6 weeks	18–65	1000 mg/day	PANSS, SANS, CGI, GAF, BACS	- The percentage of responders (patients with an improvement 20% in PANSS total score) was high in CBD group compared to placebo group, however, it could not reach statistical significance. - About 78.6% of participants improved in CBD group on CGI-I scores (CGI-I: treatment difference = 20.5, 95% CI = − 0.8, − 0.1 *p* = 0.018) compared to 54.6% in placebo arm. - CBD group had an improvement in their global functioning (treatment difference = 3.0, 95% CI = -0.4, 6.4; *p* = 0.08) and cognitive performance (treatment difference = 1.31, 95% CI = − 0.10, 2.72; *p* = 0.068), however, it could not reach statistical significance.	Mild transient GI discomfort, hyperlipidemia.	23
Zuardi et al., 2006	Schizophrenia	CBD	Case series	Level 4	3	45 days	22–23	1–5 days = Placebo 6–35 days = Participants were started on 40 mg twice a day, titrated to 1280 mg/day depending on efficacy and tolerability. 36–40 days = Placebo Last 15 days = Olanzapine	BPRS, PANSS, CGI	- Case 1: During CBD phase, symptoms improved at 1280 mg/day, followed by worsening of symptoms after CBD discontinuation. - Case 2: No improvement with CBD and partial improvement with olanzapine, requiring clozapine. - Case 3: Slight improvement with CBD. However, this patient failed to respond to olanzapine, clozapine, or haloperidol decanoate.	No side effects were reported.	24
Zuardi et al., 1995	Schizophrenia	CBD	Case report	Level 4	1	4 weeks	19	1–4 days: Placebo 4–30 days: CBD oil was increased to 1500 mg/day in divided doses. 31–34 days: Placebo After 35 days: Haloperidol was started.	BPRS	- Open BPRS scores improved from 42 to 13 and blind BPRS scores improved from 50 to 30, for an improvement of 69 and 69%, respectively. - Improvements in following factors of BPRS: thought disturbance (62.5 to 25%), hostility-suspiciousness (83.3 to 33.3%), anxiety-depression (62.5 to 18.8%), activation (58.3 to 16.7%), and anergia (31.3 to 0.0%).	No side effects were reported.	25
Zuardi et al., 2009	Psychosis in Parkinson’s disease	CBD	Open-label pilot study	Level 3	6	4 weeks	Mean age 58.8 ± 14.9 years	The initial dose of 150 mg was increased to 400 mg at the end of week 4, with an increase of 150 mg/week.	BPRS, PPQ	- There was an improvement on total scores of BPRS (*P* < 0.001) and four BPRS factors scores (Thinking disorder *p* = 0.002, Withdrawal-retardation *P* = 0.007, Anxious-depression *p* = 0.003, Activation *p* = 0.005) including positive and negative symptoms. - A reduction in scores of PPQ (*P* = 0.001) was observed at the endpoint of study.	No adverse effect on cognitive functioning was reported.	26

BACS: Brief Assessment of Cognition in Schizophrenia, BPRS: Brief Psychiatric Rating Scale, CBD: cannabidiol, CGI: Clinical Global Impressions, EPS: extrapyramidal symptoms, GAF: Global Assessment of Functioning, GI: Gastrointestinal, MCCB: MATRICS Consensus Cognitive Battery, PANSS: Positive and Negative Syndrome Scale, PPQ: Parkinson Psychosis Questionnaire, RCT: randomized controlled trial, SANS: Scale for the Assessment of Negative Symptoms, SCWT: Stroop Color Word Test, Δ9-THC: Δ9-tetrahydrocannabinol

*The Oxford Centre for Evidence-Based Medicine 2011 Levels of Evidence was used to grade the quality of evidence (OCEBM, 2019). Level 1 evidence is for systematic review of RCTs or individual RCT of narrow confidence interval, Level 2 for cohort studies or systematic review of cohort studies, Level 3 for case-control studies or systematic review of case-control studies, and Level 4 for case-series for studies focused on therapy, prevention, etiology and harm (OCEBM, 2019)

In a clinical trial, Hallak and colleagues suggested an improvement in schizophrenia-associated cognitive impairment with a CBD dose of 300 mg/day, while no significant improvement was seen at a CBD dose of 600 mg/day (Hallak et al., 2010). In another RCT, McGuire and colleagues found that CBD (1000 mg/day) improved positive psychotic symptoms, but failed to improve negative symptoms and general psychopathology associated with this illness (McGuire et al., 2018). In another RCT, Boggs and colleagues found that CBD (600 mg/day) failed to improve outcomes pertaining to reasoning and problem-solving domains (Boggs et al., 2018).

In a comparison of CBD with amisulpride, Leweke and colleagues reported similar improvements in patients taking CBD 800 mg/day and those taking amisulpride (Leweke et al., 2012). This study also indicated an increase in intrinsic anandamide signaling, an effect that explained the antipsychotic properties of CBD (Leweke et al., 2012). Moreover, CBD treatment was associated with a lower risk of extrapyramidal symptoms, less weight gain, and a lower increase in prolactin, which is a predictor of galactorrhea and sexual dysfunction (Leweke et al., 2012). An open-label study of CBD to treat psychosis in Parkinson’s disease also suggested promising results at a dose of 400 mg daily; however, there was a strong risk of bias because of inadequate blinding of participants, personnel and outcome assessors (Zuardi et al., 2009).

The remaining evidence comprised two minimal quality case reports and case series. Zuardi and colleagues were the first to report favorable findings for CBD in patients with schizophrenia (Zuardi et al., 1995). The dose of CBD ranged from 600 to 1500 mg daily in schizophrenia studies. A case series of three patients with treatment-resistant schizophrenia found improvement in only one patient (Zuardi et al., 2006). In the first case, there was an improvement in psychotic symptoms with CBD at 1280 mg/day; however, the symptoms worsened after CBD was discontinued. In second case, CBD was ineffective for the symptoms. Patient had an improvement in symptoms with clozapine. In the third case, no improvement with CBD and partial improvement with olanzapine were observed, although clozapine was subsequently required. In case 3, mild improvement was reported with CBD in a patient who had previously failed to respond to olanzapine, clozapine, or haloperidol decanoate. These results suggest a limited role of CBD in treatment-resistant schizophrenia (Zuardi et al., 2006). The dose were not individually mentioned for case 1 and 2.

Four of the included studies did not report any adverse effects of CBD among patients with psychosis. CBD was well-tolerated in these patients except for mild transient sedation, hyperlipidemia, and gastrointestinal distress. Patients with schizophrenia had fewer instances of extrapyramidal symptoms, less weight gain, and a lower increase in prolactin levels.

CBD is postulated to improve cognitive performance in psychosis through the mediation of CB_1_ and CB_2_ receptor agonism at lower concentrations (Hallak et al., 2010; Solowij et al., 2018; Manseau & Goff, 2015). This cognitive improvement has been hypothesized due to the higher concentration of cannabinoid receptors in the hypothalamus, suggesting a role in superior cognitive functioning (Hallak et al., 2010). Naturalistic studies of CBD report better cognitive performance including memory, increased grey matter in the hippocampus, and fewer psychotic symptoms in patients given higher doses of CBD (Solowij et al., 2018).

The therapeutic benefits for psychosis is hypothesized due to the inhibition of anandamide re-uptake and degradation, resulting in increased anandamide levels in the brain (Manseau & Goff, 2015). Increased anandamide levels and improvements in the symptoms of psychosis were reported in another 4-week-long RCT comparing the efficacy of CBD to amisulpride for the treatment of schizophrenia (Leweke et al., 2012). Interestingly, anandamide levels were elevated in patients with acute schizophrenia compared to chronic schizophrenia, indicating a compensatory increase in an acute state (Giuffrida et al., 2004).

#### Cannabis-related disorders

The present review included three RCTs (107 patients), two open-label trials (28 patients), one case series of four patients, and two case reports for cannabis-related disorders as summarized in Table 2 (Solowij et al., 2018; Crippa et al., 2013; Trigo et al., 2016a; Trigo et al., 2018; Trigo et al., 2016b; Allsop et al., 2014; Pokorski et al., 2017; Shannon & Opila-Lehman, 2015). Of the eight studies, level 2 evidence was found in three RCTs, level 3 evidence in two clinical trial, and level 4 evidence in two case reports and one case series (OCEBM, 2019). For cannabis-related disorders, there is Grade B recommendation based on majority of studies ranked at the level 2 and level 3 of evidence.

**Table 2:** Studies of the use of CBD and CBD-containing compounds such as nabiximols in the treatment of cannabis-related disorders and levels of evidence (1–5)

Author	Diagnosis	Study design	Pharmacological agent	Strength of evidence*	Group (n)	Duration	Age range (years)	Dose range (mg)	Scales to measure the clinical outcome	Clinical outcome	Common side effects	Reference number
Allsop et al., 2014	Cannabis withdrawal	RCT	Nabiximols	Level 2	Nabiximols = 27 Placebo = 24	6 days of nabiximols or placebo treatment, 3 days of washout, and 28-day follow-up period Total duration = 37 days	18–65	Starting dose = 8 sprays, total dose of 21.6 mg THC and 20 mg CBD at 4 PM and 10 PM Maximum dose = 8 sprays 4 times a day	CWS	- Nabiximols reduced CWS scores by 66% compared to 52% with placebo for duration for treatment (*P* = .01). - It resulted in a decrease in appetite loss, decrease in cravings (*p* = 0.4), irritability and aggression (*p* = .o1). - The time duration for cannabis withdrawal was 3.10 days with Nabiximols compared to 4.9 days with placebo (*p* = .04) - The retention rate was 85% with medications compared to 50% with placebo.	Decreased appetite. The number and severity of adverse events did not differ significantly between groups.	31
Trigo et al., 2016	Cannabis moderate-severe use and withdrawal	RCT	Nabiximols	Level 2	16	8 weeks	18–50	Nabiximols = 108 mg THC/100 mg CBD Fixed dose = 4 sprays of medications every hour Self-titrated dose: Patients were allowed to use 4 sprays as needed every hour. The maximum dose was 40 sprays/day.	MCQ, CWS, SMHSQ, DEQ, ARCI, MNWS	- Medication intake was higher on fixed regimen as compared to self-titration conditions. There was significant differences between conditions (F(3,24) = 8.561, *p* < 0.001). - Mean time for having feeling of “high” was clearly higher during SAU (6.6–7.3 h) compared with Sativex (2.4–3.3 h) or placebo (0.1–0.3 h), as self-reported by participants in their smoking diary (Fig. 1c) - There were lesser withdrawal during self-titrated and fixed Sativex as compared to the corresponding placebo conditions (F(7,56) = 3.860, *p* < 0.01).	No significant difference in side effects was observed between the experimental and placebo group.	28
Trigo et al., 2018	Cannabis moderate to severe use	RCT	Nabiximols	Level 2	Nabiximols and weekly MET/CBT = 20 Placebo = 20	12 weeks	18–65	Nabiximols = 113.4 mg THC/105 mg CBD	BPRS, SAFTEE, HAM-A, HDRS, TLFB for cannabis, tobacco, caffeine and alcohol, FTND, ASI, BDI, DEQ, Profile of Mood States, MWC, MCQ-SF, SMHSQ	- Nabiximols was well-tolerated with a dose range of 4.1 to 12.8 sprays/day. - There was reduction in cannabis use in the nabiximols (70.5%) and placebo groups (42.6%). - Five participants in the placebo group and four participants in the nabiximols group used other recreational drugs. - High medication sub-group suggested a significant effect of time (F12,90.1 = 10.386, *p* < .001), no effects of treatment (F1,8.1 = 1.200, *p* = .305) but a significant time x treatment interaction.	No serious adverse events were observed.	29
Pokorski et al., 2017	Cannabis withdrawal	Open-label pilot study	CBD	Level 3	8	7 days	21–62 Mean age = 40 years	CBD = 600–1200 mg/day in divided doses	CWS, daily urine sample and blood samples on day 1, 3, and 7, THC COOH and CBD quantification	- For 600 mg/day of CBD: 2 out of 5 participants completed the 7-day inpatient treatment. These 2 participants reported abstinence at follow-up (day 28) and the 3 remaining participants reported decreased cannabis use, confirmed by blood and urine analysis. - For 600 mg twice a day: of 3 participants, 2 reported abstinence and the 1 remaining one had decreased use of cannabis, confirmed by blood and urine analysis. - All participants reported a decrease in CWS score.	The participants did not report any unwanted or adverse effects of the CBD.	32
Solowij et al., 2018	Impaired cognition and elevated psychological symptoms in patients with chronic cannabis use	Open-label trial	CBD	Level 3	20	10 weeks	Median age = 25.1 years	CBD = 200 mg in divided doses	BDI, STAI-I, STAI-II, GAF, SOFAS, CAPE, RAVLT, AST	- There was an improvement in severity of depression (*p* = 0.017), verbal learning, memory performance, and frequency of positive psychotic-like symptoms (*p* = 0.025) with decreased level of distress from baseline to endpoint. - The state anxiety increased with no change in trait anxiety, functional impairment, and accuracy on cognitive tests.	No side effects were reported.	19
Trigo et al., 2016	Cannabis moderate to severe use	Case series	Nabiximols	Level 4	4	12-week follow-up phase with 4 weekly visits and 2 subsequent monthly visits	24–43 Mean age = 35 years	Self-titrated nabiximols = 77.5–113.4 mg THC 71.5–105 mg CBD	CWC, CCQ, TLFB for cannabis, tobacco, caffeine and alcohol	- Reduction in cannabis intake from baseline to endpoint with no compensatory increase in use of other substances (F(18,54) = 4.663, p < 0.001). - The craving scores increased initially during the first 2 weeks with a subsequent reduction in craving from week 9 (F(18,54) = 7.091, *p* < 0.001) . - No significant difference in withdrawal scores for the duration of study (F(18,54) = 0.805, *p* value = non-significant)	No side effects were reported.	30
Crippa et al., 2013	Cannabis withdrawal syndrome	Case report	CBD	Level 4	1	10 days	19	The dose of CBD was 300 mg on day 1 and 600 mg on days 2–10. 600 mg was administered in divided doses.	MWC, WDS	- CBD resulted in faster, progressive relief from withdrawal, anxiety, and dissociative symptoms. - Marijuana withdrawal symptom checklist had drop of baseline score of 12 to zero, from 5 to zero for Withdrawal discomfort scale. - The scores for Beck Anxiety Inventory decreased from 6 to zero and 10 to zero for Beck Depression Inventory. - At 6 month follow-up, return to cannabis use but at a lower rate.	No side effects were reported.	27
Shannon & Lehman, 2015	Cannabis moderate to severe use	Case report	CBD	Level 4	1	Follow-up for 129 days	27	Initial regimen: 24 mg CBD (6 sprays as needed during the day and 2 sprays at night). The dose was decreased to 18 mg with 6 spray at night only.	Self-reported cannabis use, PSQI, HAM-A	- Patient was able to maintain abstinence from cannabis. - Improvement in HAM-A score from 16 to 8 was reported, indicating mild anxiety. - Patient had a regular sleep schedule and scores of 7 to eight were reported.	No side effects were reported.	33

ARCI: Addiction Research Center Inventory, ASI: Addiction Severity Index, AST: Attention Switching Task, BDI: Beck Depression Inventory, BPRS: Brief Psychiatric Rating Scale, CAPE: Community Assessment of Psychic Experiences-Positive Scale, CBD: cannabidiol, CBT: cognitive–behavioral therapy, CCQ: Cannabis Craving Questionnaire, CWS: Cannabis Withdrawal Scale, DEQ: Drug Effects Questionnaire, FTND: Fagerstrom Test for Nicotine Dependence, GAF: Global Assessment of Functioning, HAM-A: Hamilton Anxiety Rating Scale, HDRS: Hamilton Rating Scale for Depression, MCQ: Marijuana Craving Questionnaire, MCQ-SF: Marijuana Craving Questionnaire-Short Form, MET: motivational enhancement therapy, MNWS: Minnesota Nicotine Withdrawal Scale, MWC: Marijuana Withdrawal Symptom Checklist, PSQI: Pittsburgh Sleep Quality Index, RAVLT: Rey Auditory Verbal Learning Test, SAFTEE: Systematic Assessment for Treatment Emergent Events, SOFAS: Social and Occupational Functioning Assessment Scale, SMHSQ: St Mary’s Hospital Sleep Questionnaire, STAI: Spielberger State-Trait Anxiety Inventory, TLFB: Timeline Follow-Back, WDS: Withdrawal Discomfort Score, THCCOOH: 11-nor-9-carboxy-Δ9-tetrahydrocannnabinol, Δ9-THC: Δ9-tetrahydrocannabinol

“The Oxford Centre for Evidence-Based Medicine 2011 Levels of Evidence was used to grade the quality of evidence (OCEBM, 2019). Level 1 evidence is for systematic review of RCTs or individual RCT of narrow confidence interval, Level 2 for cohort studies or systematic review of cohort studies, Level 3 for case-control studies or systematic review of case-control studies, and Level 4 for case-series for studies focused on therapy, prevention, etiology and harm (OCEBM, 2019)

Four of these studies evaluated the efficacy of nabiximols, and four others reported the use of CBD. The doses tested ranged from 20 mg CBD to a maximum of 1200 mg/day. Nabiximols was used in spray form at doses ranging from an average of 28.9 sprays/day (equivalent to 77.5 mg THC or 71.7 mg CBD) to 40 sprays/day (equivalent to 108 mg THC or 100 mg CBD). In CBD-only studies the dose of CBD ranged from 200 to 600 mg/day in divided doses. All three RCTs in this section provided evidence for the use of nabiximols for moderate to severe cannabis use disorder. These trials tested different doses of nabiximols ranging from 21.6 mg THC and 20 mg CBD (twice a day) to 113.4 mg THC or 105 mg CBD per day. All trials reported lower withdrawal rates, better tolerance, and retention rates in the experimental group. Moreover, no serious adverse effects were reported in any of these studies. In one RCT, nabiximols (total dose of 21.6 mg THC and 20 mg CBD at 4 and 10 in evening and night, respectively) was associated with marked improvement in cannabis withdrawal symptoms, leading to shorter withdrawal times and higher retention rates (Allsop et al., 2014). In a second RCT, a fixed dose of nabiximols produced more positive results compared to self-titrated administration (Trigo et al., 2016a). Patients in the fixed-dose group had four sprays of medications every hour compared to four sprays as needed every hour in self-titrated dose group. The maximum dose was 40 sprays/day in the self-titrated dose group. Medication intake was higher with fixed doses, which were associated with fewer withdrawal symptoms compared to the self-titrated regimen (Trigo et al., 2016a). In another RCT, the efficacy and safety of nabiximols were compared to a placebo while all participants also received weekly motivational enhancement therapy (MET) and cognitive–behavioral therapy (CBT) (Trigo et al., 2018). The dose range of 4.1 to 12.8 sprays/day was reported among nabiximols group. The withdrawal scores in this study were similar in both groups (Trigo et al., 2018). Only one of the studies reported decreased appetite, while the number and severity of adverse effects were not reported or observed in the other two RCTs.

Two open-label studies testing the effectiveness of two different concentrations of CBD (200 mg/day and 600–1200 mg/day) obtained positive outcomes with doses as low as 600 mg/day (Hallak et al., 2010; Pokorski et al., 2017). These studies had a small sample size of eight (Solowij et al., 2018) and 20 (Pokorski et al., 2017) participants, respectively. In the former open-label trial with eight participants, a dose of 600 mg/day was tested, and two out of five participants completed the 7-day inpatient treatment. These two participants reported abstinence at follow-up (day 28), and the remaining three participants reported decreased use of cannabis, confirmed by blood and urine analysis. In the second group, participants took 600 mg twice a day. Two out of three participants reported abstinence and in the remaining one, cannabis use had decreased, as confirmed by blood and urine analysis. All participants showed a decrease in Cannabis Withdrawal Scale scores. The second open-label trial tested the effectiveness of 200 mg CBD in divided doses in improving cognition and depressive symptomatology among patients with chronic cannabis use, and found improvement in severity of depression, verbal learning, and memory performance, and decreased frequency of positive psychotic-like symptoms and level of distress from baseline to endpoint (Solowij et al., 2018). State anxiety increased with no change in trait anxiety, functional impairment, or accuracy on cognitive tests (Solowij et al., 2018).

The remaining studies were either case series or case reports; all found positive outcomes in withdrawal and cannabis-dependence symptoms (Crippa et al., 2013; Trigo et al., 2016b; Shannon & Opila-Lehman, 2015). Mean age in the case series was 35 years, although the first participant was 19 years old and the second was 27 years old. The case series used self-titrated nabiximols at a dose of 77.5–113.4 mg THC and 71.5–105.0 mg CBD (Trigo et al., 2016b). Moreover, all participants reported a significant reduction in craving (Crippa et al., 2013; Trigo et al., 2016b; Shannon & Opila-Lehman, 2015), quicker relief (Crippa et al., 2013), lower anxiety, and an improved sleep schedule (Shannon & Opila-Lehman, 2015). However, the case series reported increased craving scores during the first 2 weeks with a subsequent reduction in craving at week 9. CBD was well-tolerated in this patient population, except for decreased appetite reported in one study (Trigo et al., 2016b). For patients receiving nabiximols or CBD, treatment should be augmented with psychotherapeutic modalities considering the positive evidence for an effect on cravings.

The effectiveness and tolerability of CBD and nabiximols for moderate to severe cannabis use disorder was reported in several studies. The efficacy may also be due to the synergetic or additive benefits of Δ9-THC and CBD rather than CBD alone. The Δ9-THC component of nabiximols decreases the severity of withdrawal symptoms, lowering the risk of relapse (Trigo et al., 2016a). However, there is mixed evidence for the role of nabiximols in cannabis-related craving (Trigo et al., 2016a; Trigo et al., 2018; Trigo et al., 2016b). Studies that included combined motivation enhancement and behavioral response prevention strategies suggested a reduction in craving (Trigo et al., 2016a; Trigo et al., 2018). CBD is thought to modulate the euphoric, anxiogenic, psychological, and physiological effects of Δ9-THC (Crippa et al., 2013). However, these benefits of CBD alone and in combination with THC need to be explored in head-to-head studies.

#### Other disorders

The present review included two RCTs (54 patients), one open-label trial (53 patients), one retrospective chart review (72 patients), and four case reports for CBD and nabiximols use in the treatment of other psychiatric disorders. Specifically, this review looked at ADHD (one RCT), comorbidities in ASD (one open-label trial), anxiety and sleep problems (one retrospective chart review), SAD (one clinical trial), bipolar disorder (one case report), PTSD (one case report), and Tourette syndrome (two case reports), as summarized in Table 3 (Cooper et al., 2017; Barchel et al., 2018; Bergamaschi et al., 2011; Shannon et al., 2019; Zuardi et al., 2010; Shannon & Opila-Lehman, 2016; Trainor et al., 2016; Pichler et al., 2019). Of the nine studies, level 2 evidence was found in two RCTs, level 3 evidence in one clinical trial, and level 4 evidence in one retrospective chart review, four case reports (OCEBM, 2019). There is Grade B recommendation for comorbidities in patients with ASD, anxiety disorders including SAD and sleep problems, and ADHD where as bipolar disorder, PTSD and Tourette Syndrome has Grade C recommendation. However, this should be considered in the context of fewer studies of each these diagnoses.

**Table 3:** Studies of the use of CBD and CBD-containing compounds such as nabiximols in the treatment of other psychiatric disorders and levels of evidence (1–5)*

Author	Diagnosis	Study design	Pharmacological agent	Strength of evidence	Group (n)	Duration	Age range (years)	Dose range (mg)	Scales to measure the clinical outcome	Clinical outcome	Common side effects	Reference number
Cooper et al., 2017	ADHD	RCT	Nabiximols	Level 2	Nabiximols = 15 Placebo = 15	6 weeks	18–55	Nabiximols oromucosal spray = 2.7 mg Δ9-THC and 2.5 mg CBD Mean dose = 4.7 sprays per day Maximum dose = 14 sprays/day	QbTest	- The experimental group had better scores compared to placebo group (Est = 0.17, 95%CI-0.40 to 0.07, *p* = 0.16, *n* = 15/11 active/placebo). - Nabiximols was associated with a nominally significant improvement in hyperactivity/impulsivity (*p* = 0.03) and a cognitive measure of inhibition (*p* = 0.05), and a trend towards improvement for inattention (*p* = 0.10) and executive learning (*p* = 0.11).	Muscular seizures and spasms	34
Barchel et al., 2018	ASD and related comorbidities 1. Hyperactivity 2. Sleep problems 3. Self-injury 4. Anxiety	Open-label trial	CBD and Δ9-THC	Level 3	53	30–588 days Median duration = 66 days	4–22 Median age = 11 years	CBD oil solution with CBD and Δ9-THC at 1:20 ratio CBD 16 mg/kg (maximal daily dose 600 mg) CBD median IQR daily dose = 90 (45–143) mgΔ9-THC 0.8 mg/kg (maximal daily dose 40 mg). THC median IQR daily dose = 7 (4–11) mg	Not mentioned	- These patients were taking concomitant medications including stimulants, antipsychotics, anti-epileptics, melatonin, anti-depressants, alpha-agonists, and anti-muscarinic agents. - Out of 53 patients, 74.5% reported improvement in comorbid symptoms. - About 68.4% reported improvement in hyperactivity, 67.6% in self-injurious behaviors, 71.4% in sleep problems, and 47.1% in anxiety symptoms.	Somnolence and change in appetite	35
Bergamasci et al., 2011	Anxiety related to public speaking	RCT	CBD	Level 4	CBD = 12 Placebo = 12	Single dose	SAD-placebo = 22.8 SAD-CBD = 24.6 Healthy = 23.3	600 mg	Mini-SPIN, VAMS, SSPS, SSPS-N, BSS	- Pretreatment with a single dose of CBD significantly decreased anxiety, cognitive impairment and discomfort in speech performance. It also resulted in significantly decreased alertness in anticipatory speech. - There were significant effect of phases (F3.6,118.51/432.7; *p* < 0.001), group (F2,331/413.5; *p* < 0.001) and phases by group interaction (F7.2,118.5 1/4 6.4; p < 0.001). - There were also significant differences between placebo and healthy control group at the initial (*p* < 0.018), anticipatory (p < 0.001), speech (p < 0.001) and post-speech (0.018) phases. - The CBD group differs from the placebo (*p* < 0.012) and control (*p* < 0.007) groups during the speech phase	No side effects were reported.	36
Shannon et al., 2019	Anxiety and insomnia	Retrospective chart review	CBD	Level 4	Anxiety = 47 Sleep disorder = 25	3 months	Sleep disorder = 18–72 Mean age = 36.5 years Anxiety = 18–70 Mean age = 34 years	25–175 mgMost patients received 25 mg/day	HAM-A, PSQI	- Most patients received 25 mg/day CBD; a handful of patients received 50 or 75 mg/day. One patient with schizoaffective disorder and trauma was given up to 175 mg/day. - After 1 month of treatment, 79.2 and 66.7% of patients reported improvement in anxiety and sleep, respectively. - After 2 months, 78.1 and 56.1% of patients reported improvement in anxiety and sleep, respectively, which were also observed at 3-month follow-up. - Greater improvement in anxiety scores than sleep scores.	- 2 patients discontinued treatment due to fatigue and 1 patient with a development disorder had increased sexually inappropriate behaviors, resulting in discontinuation - Transient mild sedation was also reported in some patients.	37
Zuard et al., 2010	Bipolar disorder	Case report	CBD	Level 4	2	38 days	34 and 36	1–5 days for both participants: Placebo Case 1: 5–19 days: CBD 600 mg and olanzapine 10–15 mg 20–33 days: CBD 900–1200 mg Case 2: 5–33 days: CBD 600–1200 mg 33–38 days: Placebo	BPRS, YMRS	- Case 1: 37 and 33% improvement on BPRS and YMRS with CBD and olanzapine, but no additional improvement with CBD monotherapy. - Case 2: CBD failed to improve symptoms of bipolar disorder at any of the prescribed doses.	No side effects were reported.	38
Shannon & Opila-Lehman., 2016	Posttraumatic stress disorder	Case report	CBD	Level 4	1	5 months of CBD	10	CBD oil 25 mg Liquid CBD 6–12 mg sublingual spray as needed for anxiety	SDSC, SCARED	- CBD scores improved from 34 to 18 at endpoint, indicating no anxiety. - Sleep Disturbance Scale scores improved from 59 to 38, suggesting no problem with sleep.	No side effects were reported.	39
Trainor et al., 2016	Tourette syndrome	Case report	Nabiximols	Level 4	1	4 weeks	26	Two oromucosal sprays of nabiximols BID Total dose = 10.8 mg Δ9-THC, 10 mg CBD per day	YGTSS, ORVRS	- Using the ORVRS to evaluate tics, motor tics were reduced by 85% and vocal tics by 90% - Number of affected body areas decreased. - mprovement of 35% on YGTSS.	No side effects were reported.	40
Pichler et al., 2018	Tourette syndrome	Case report	Cannabis tincture THC combined with CBD	Level 4	1	2 months	47	34 drops cannabis tincture 3 times a day = 10 mg Δ9-THC combined with 20 mg of CBD	YGTSS	- With the combination of Δ9-THC and CBD, there was significant improvement in tic frequency and severity. - Scores decreased from 73/100 to 44/100 on YGTSS. - Patient reported improvement in quality of life and enhanced social activity.	Slight xerostomia	41

ADHD: Attention-deficit/hyperactivity disorder, ASD: Autism spectrum disorder, BPRS: Brief Psychiatric Rating Scale, BSS: Bodily Symptoms Scale, CBD: cannabidiol, HAM-A: Hamilton Anxiety Rating Scale, IQR: Interquartile range, Mini-SPIN: Mini-Social Phobia Inventory, ORVRS: Original Rush Videotape Rating Scale, PSQI: Pittsburg Sleep Quality Index, QbTest: Quantified Behavioral Test, RCT: randomized controlled trial, SCARED: Screen for Anxiety Related Disorders, SDSC: Sleep Disturbance Scale for Children, SSPS: Self-Statements During Public Speaking, SSPS-N: Negative Self-Statements, VAMS: Visual Analog Mood Scales, YGTSS: Yale Global Tic Severity Scale, YMRS: Young Mania Rating Scale, Δ9-THC: Δ9-tetrahydrocannabinol

*The Oxford Centre for Evidence-Based Medicine 2011 Levels of Evidence was used to grade the quality of evidence (OCEBM, 2019). Level 1 evidence is for systematic review of RCTs or individual RCT of narrow confidence interval, Level 2 for cohort studies or systematic review of cohort studies, Level 3 for case-control studies or systematic review of case-control studies, and Level 4 for case-series for studies focused on therapy, prevention, etiology and harm (OCEBM, 2019)

The oromucosal nabiximols spray was tested to evaluate its effects on cognitive performance, hyperactivity, inattention, and emotional lability in 15 participants in a placebo-controlled RCT (Cooper et al., 2017). The mean dose of nabiximols was 4.7 sprays per day (2.7 mg Δ9-THC and 2.5 mg CBD). Although an improvement in these symptoms was observed in the intervention group, it failed to reach statistical significance (Cooper et al., 2017). However, this result may not be valid or reliable due to the low power of the study.

One case report on the use of CBD by two patients with bipolar disorder showed limited to no improvement with doses of 600–1200 mg for bipolar mania in one of the patients (Shannon et al., 2019). The second patient was prescribed CBD 600 mg (5–9 days) and olanzapine (10–15 mg), followed by CBD 900–1200 mg (20–33 days), and showed improvement on the Brief Psychiatric Rating Scale (37% reduction) and Young Mania Rating Scale (33% reduction) with CBD and olanzapine, but no additional improvement with CBD monotherapy (Shannon et al., 2019). This effect was consistent with results from animal studies that modeled acute mania with dextroamphetamine (Shannon et al., 2019). The lack of effectiveness can be attributed to the shorter duration of treatment in both cases. This evidence from studies of bipolar mania should be considered in the context of different pharmacological agents responding differently to certain episodes of bipolar disorder. In animal studies, CBD induced a rapid, persistent antidepressant response by increasing brain-derived neurotrophic factor in the prefrontal cortex (Shannon et al., 2019). Given its possible antidepressant benefits, the role of CBD should be explored in unipolar and bipolar depression.

In an open-label trial involving children with ASD, Barchel and colleagues reported that a solution of CBD and Δ9-THC (1,20 ratio) was effective for hyperactivity, insomnia, self-injurious behaviors, and anxiety (Barchel et al., 2018). The median dose was 90 mg with an interquartile range (IQR) of 45–143 mg for CBD whereas The medical dose was 7 mg with IQR of 4–11 mg. In this cohort of 53 patients, 74.5% showed improvement in their comorbid symptoms, 68.4% in hyperactivity, 67.6% in self-injurious behaviors, 71.4% in sleep problems, and 47.1% in anxiety symptoms. This treatment regimen lasted for a median of 66 days. However, Salgado and Castellanos suggested guiding principles for the use of CBD in this population, including a better clinical understanding of CBD, open discussion with parents and patients, addressing their perceptions, promoting informed consent, and exercising caution in the use of CBD (Salgado & Castellanos, 2018). Patients with ASD make up a heterogeneous group of individuals with different comorbidities that should be considered.

The efficacy of CBD for SAD and PTSD was explored in three studies including one RCT, one case report, and one chart review. The RCT reported the results of a simulated public speaking test among 12 healthy control participants and 24 patients with SAD who received a single dose of CBD 600 mg or a placebo before the test. This study reported that pretreatment with CBD resulted in less anxiety, cognitive impairment, and discomfort during their speaking performance. It also resulted in a significant reduction in alertness in their anticipatory speech compared to the placebo group (Bergamaschi et al., 2011).

In a 10-year-old patient, 5 months of treatment with CBD oil (25 mg) and liquid CBD (6–12 mg) in a sublingual spray as needed was associated with less anxiety and better sleep quality, with no adverse effects (Shannon & Opila-Lehman, 2016). These results were replicated for anxiety in a recently published chart review of 72 adult patients with insomnia and anxiety (Shannon et al., 2019). Most patients in this group were given 25 mg CBD/day, while a few patients were given 50 or 75 mg/day, and one patient with schizoaffective disorder and trauma was given up to 175 mg/day. All patients showed less anxiety and improved sleep, with reductions of 65–80% in the Hamilton Anxiety Rating Scale and Pittsburgh Sleep Quality Index scores.

Nabiximols produced improvements in patients with Tourette syndrome at a much lower dose than what was used for cannabis-related disorders (Trainor et al., 2016; Pichler et al., 2019). These case reports tested two oromucosal nabiximols sprays used twice a day (total dose 10.8 mg Δ9-THC and 10 mg CBD per day) (Trainor et al., 2016), and the second also tested cannabis tincture (34 drops three times a day (Pichler et al., 2019). Both case reports found improvements in tic frequency (Trainor et al., 2016; Pichler et al., 2019), severity (Trainor et al., 2016; Pichler et al., 2019), quality of life, and social activity (Trainor et al., 2016). These treatments regimens were used for 4 weeks with the oromucosal spray form (Trainor et al., 2016) and 8 weeks for cannabis tincture (Pichler et al., 2019). The therapeutic benefits can be attributed to the anxiolytic and sleep-inducing properties of CBD (Trainor et al., 2016). It is difficult to ascertain whether these improvements were due to due to CBD, Δ9-THC, additive, or synergetic effects. The anxiolytic properties of CBD explain the attenuation of anxiety associated with the onset of tics, and the improvement in tics with a combination of Δ9-THC and CBD (Trainor et al., 2016; Pichler et al., 2019).

Adverse effects were reported in four of the studies, and included muscular seizures and spasms (Cooper et al., 2017), somnolence and changes in appetite (Barchel et al., 2018), fatigue, and sexually inappropriate behavior in a patient with developmental disorder (Shannon et al., 2019), mild sedation (Zuardi et al., 2010), and mild xerostomia (Pichler et al., 2019).

### Summary of evidence

The present article provides a comprehensive review of the evidence supporting the use of CBD and CBD-containing compounds such as nabiximols to treat psychiatric disorders. CBD and nabiximols were effective in cannabis use-related disorders, and preliminary evidence was found in support of their use for other psychiatric disorders. Of the 23 studies reviewed here, level 2 evidence was found in eight RCTs, level 3 evidence in four open-label trials and one clinical trial, and level 4 evidence in one retrospective chart review, seven case reports, and two case series, according to the Oxford Centre for Evidence-Based Medicine 2011 Levels of Evidence (OCEBM, 2019). This review covers the evidence for different routes of administration, e.g. oral, inhalation spray, and sublingual. The bioavailability of these routes (11–13% for oral vs. 11–43% for inhalation) varies significantly – a factor that can impact the efficacy of different formulations.

Their antipsychotic, neuroprotective, anxiolytic, and sedating properties suggest a potential therapeutic role of CBD and nabiximols to treat various psychiatric disorders. The use of CBD at higher doses (above 1200 mg per day) showed promising results in case studies of schizophrenia and psychosis in patients with Parkinson’s disease, except in treatment-resistant cases. Regarding the use of CBD to treat anxiety disorders, its anxiolytic effect can help patients with PTSD-related and social performance-related anxiety, and nabiximols can reduce the anxiety associated with the onset of tics. There is also favorable evidence in patients with ASD for reducing hyperactivity, self-injurious behaviors, anxiety, and insomnia. Nabiximols showed no credible effect in the treatment of ADHD, while CBD was also found to be ineffective for bipolar disorder. Of all the cases examined, the strongest evidence was found for the treatment of cannabis-related disorders. The use of nabiximols yielded positive results in multiple studies of moderate to severe cannabis use disorder; however, the use of CBD alone has not been adequately documented outside a few cases and case series. Notably, CBD compounds were helpful in alleviating psychotic symptoms and improving cognitive impairment in patients across a variety of conditions.

### Recommendations for future research

This review found low-level evidence for the use of cannabis and nabiximols in a variety of disorders. Despite our comprehensive literature search, only a few RCTs related to the disorders of interest were found. These RCTs were marred by a number of limitations, most importantly failure to blind the outcome assessor, participants, and research personnel (in the open-label trials). In addition, most RCTs had a small sample size, critically reducing the power of the study to draw robust conclusions. The findings of the RCTs reviewed here need to be validated via a series of larger, well planned, randomized, double-blinded, and placebo-controlled studies. The present report can be used to design and plan further studies; however, at present the use of CBD and nabiximols in clinical practice cannot be recommended with confidence due to the drawbacks noted above.

The evidence from studies included in this review can guide future trials by providing information pertaining to the dosages, formulations and routes of administration of CBD and nabiximols. Moreover, future studies should investigate different routes of administration in light of the differences in bioavailability. In view of the (albeit limited) evidence for treatment-resistant schizophrenia, the role of CBD should be explored in the early stages of psychosis or as an adjunct medication. Although CBD was ineffective for bipolar mania, its possible efficacy as an antidepressant should be assessed in studies focused on bipolar depression. Nabiximols has been helpful in cannabis-related disorder and Tourette syndrome, owing to the synergetic benefits of CBD and THC. Future studies designed to explore the comparative benefits of these treatments can shed further light on their clinical potential. Future RCTs should also consider adding first-line treatment agents as comparison arms, to ascertain the comparative efficacy of CBD in different mental disorders. Although fewer side effects were reported overall by patients in the studies reviewed here, the vulnerability to addiction to cannabinoids should not be ignored.

### Limitations of the review

This review article has several limitations that should be considered. This review article provides evidence for CBD and CBD-containing nabiximols are two different pharmacological agents. Nabiximols has two active compounds and included studies do not consider the separate effects of THC VS CBD. There is need for future analyses to carefully consider their benefits individually. Only one-third of studies (8/23) in this review article are RCTs and most of these RCTs had a small sample size decreasing the power of the study to draw robust conclusions.

## Conclusion

The evidence reviewed here favors CBD use for patients with schizophrenia and psychosis in Parkinson’s disease in four out of seven studies, except in treatment-resistant cases. There is a Grade B recommendation this diagnosis based on the levels of evidence. Nabiximols and CBD were beneficial in cannabis-related disorders in almost all studies with Grade B recommendation, resulting in a decreased risk of withdrawal symptoms and dependence among participants. The effect on cannabis-related craving was pronounced, with an additive benefit from the use of psychotherapeutic options such as MET or CBT. One open-label trial suggested favorable evidence for the use of cannabinoids CBD and Δ9-THC for hyperactivity, self-injurious behaviors, and anxiety symptoms in patients with ASD with Grade B recommendation. CBD was helpful in patients with anxiety and insomnia related to SAD and PTSD in one chart review. Nabiximols was found to be effective in reducing the frequency and severity of tics and improving the quality of life in patients with Tourette syndrome according to case reports. There was no firm evidence to support CBD to treat bipolar mania (one case report) or nabiximols (one RCT) to treat ADHD. There is Grade B (moderate) recommendation for attention deficit hyperactivity disorder. Grade C recommendation (weaker) exists for insomnia, anxiety, bipolar disorder, posttraumatic stress disorder, and Tourette syndrome. These recommendations should be considered in the context of limited number of available studies. The authors recommend well-planned randomized controlled trials to further study the benefits of CBD and CBD-containing options such as nabiximols in patients with psychiatric disorders. It is also important to assess the individual pharmacodynamic and pharmacokinetic effects of CBD and Δ9-THC in different treatments.

## Data Availability

Available to others on request.

## Abbreviations

5-HT: 5-hydroxytryptamine
ADHD: Attention deficit hyperactivity disorder
ARCI: Addiction Research Center Inventory
ASD: Autism spectrum disorder
ASI: Addiction Severity Index
AST: Attention Switching Task
AUC: Area Under Curve
BACS: Brief Assessment of Cognition in Schizophrenia
BDI: Beck Depression Inventory
BDNF: Brain-derived neurtrophic factor
BPRS: Brief Psychiatric Rating Scale
BSS: Bodily Symptoms Scale
CAPE: Community Assessment of Psychic Experiences-Positive Scale
CB1 receptor: Cannabinoid receptor 1
CB2 receptor: Cannabinoid receptor 2
CBD: Cannabidiol
CBT: Cognitive–behavioral therapy
CCQ: Cannabis Craving Questionnaire
CGI: Clinical Global Impression
Cmax: Maximum Serum Concentration
CSF: Cerebrospinal fluid
CWS: Cannabis Withdrawal Scale
CYP: Cytochrome P450
DEQ: Drug Effects Questionnaire
EPS: Extrapyramidal symptoms
FTND: Fagerstrom Test for Nicotine Dependence
GAF: Global Assessment of Functioning
GI: Gastrointestinal
GPR: G-protein-coupled receptor
HAM-A: Hamilton Anxiety Rating Scale
HDRS: Hamilton Rating Scale for Depression
IQR: Interquartile range
MCCB: MATRICS Consensus Cognitive Battery
MCQ: Marijuana Craving Questionnaire
MCQ-SF: Marijuana Craving Questionnaire-Short Form
MET: Motivational Enhancement Therapy
Mini-SPIN: Mini-Social Phobia Inventory
MNWS: Minnesota Nicotine Withdrawal Scale
MWC: Marijuana Withdrawal Symptom Checklist
OCEBM: Oxford Centre for Evidence-based Medicine – Levels of Evidence
ORVRS: Original Rush Videotape Rating Scale
PANSS: Positive and Negative Syndrome Scale
PPQ: Parkinson Psychosis Questionnaire
PSQI: Pittsburgh Sleep Quality Index
PTSD: Post-traumatic Stress Disorder
QbTest: Quantified Behavioral Test
RAVLT: Rey Auditory Verbal Learning Test
RCT: Randomized controlled trial
SAD: Social Anxiety Disorder
SAFTEE: Systematic Assessment for Treatment Emergent Events
SANS: Scale for the Assessment of Negative Symptoms
SCARED: Screen for Anxiety-related Disorders
SCWT: Stroop Color Word Test
SDSC: Sleep Disturbance Scale for Children,
SMHSQ: St Mary’s Hospital Sleep Questionnaire
SOFAS: Social and Occupational Functioning Assessment Scale
SSPS: Self-Statements During Public Speaking
SSPS-N: Negative Self-Statements
STAI: Spielberger State-Trait Anxiety Inventory
THC: Tetrahydrocannabinol
THCCOOH: 11-nor-9-carboxy-Δ9-tetrahydrocannnabinol
TLFB: Timeline Follow-Back
TRPV: Transient receptor potential channels
UDP-glucuronosyltransferases: Uridine 5′-diphospho-glucuronosyltransferase
USA: United States of America
VAMS: Visual Analog Mood Scales
WDS: Withdrawal Discomfort Score
YGTSS: Yale Global Tic Severity Scale
YMRS: Young Mania Rating Scale
Δ9-THC: Δ9-tetrahydrocannabinol
